# Geographical structure, narrow species ranges, and Cenozoic diversification in a pantropical clade of epiphyllous leafy liverworts

**DOI:** 10.1002/ece3.2656

**Published:** 2016-12-20

**Authors:** Julia Bechteler, Alfons Schäfer‐Verwimp, Gaik Ee Lee, Kathrin Feldberg, Oscar Alejandro Pérez‐Escobar, Tamás Pócs, Denilson F. Peralta, Matthew A. M. Renner, Jochen Heinrichs

**Affiliations:** ^1^ Department of Biology I, Systematic Botany and MycologyGeoBio‐CenterUniversity of Munich (LMU)MunichGermany; ^2^ Herdwangen‐SchönachGermany; ^3^ School of Marine and Environmental SciencesUniversity of Malaysia TerengganuKuala TerengganuTerengganuMalaysia; ^4^ Botany DepartmentEszterházy UniversityEgerHungary; ^5^ Instituto de BotânicaSão PauloSPBrazil; ^6^ Royal Botanic Gardens and Domain TrustSydneyNSWAustralia

**Keywords:** ancestral area estimation, bryophyte, cryptic speciation, divergence time estimation, epiphyte, *Leptolejeunea*, phylogeny

## Abstract

The evolutionary history and classification of epiphyllous cryptogams are still poorly known. *Leptolejeunea* is a largely epiphyllous pantropical liverwort genus with about 25 species characterized by deeply bilobed underleaves, elliptic to narrowly obovate leaf lobes, the presence of ocelli, and vegetative reproduction by cladia. Sequences of three chloroplast regions (*rbc*L, *trn*L‐F, *psb*A) and the nuclear ribosomal ITS region were obtained for 66 accessions of *Leptolejeunea* and six outgroup species to explore the phylogeny, divergence times, and ancestral areas of this genus. The phylogeny was estimated using maximum‐likelihood and Bayesian inference approaches, and divergence times were estimated with a Bayesian relaxed clock method. *Leptolejeunea* likely originated in Asia or the Neotropics within a time interval from the Early Eocene to the Late Cretaceous (67.9 Ma, 95% highest posterior density [HPD]: 47.9–93.7). Diversification of the crown group initiated in the Eocene or early Oligocene (38.4 Ma, 95% HPD: 27.2–52.6). Most species clades were established in the Miocene. *Leptolejeunea epiphylla* and *L. schiffneri* originated in Asia and colonized African islands during the Plio‐Pleistocene. Accessions of supposedly pantropical species are placed in different main clades. Several monophyletic morphospecies exhibit considerable sequence variation related to a geographical pattern. The clear geographic structure of the *Leptolejeunea* crown group points to evolutionary processes including rare long‐distance dispersal and subsequent speciation. *Leptolejeunea* may have benefitted from the large‐scale distribution of humid tropical angiosperm forests in the Eocene.

## 1 Introduction

Range estimation is a challenging theme in morphologically little differentiated groups of organisms and suitable to improve understanding of species diversity and evolution. Many bryophyte genera belong to these critical groups and are in need of thorough reinvestigation including integrative molecular–morphological approaches; however, to date, only a limited number of studies is available (Dong et al., 2012; Forrest, Salazar‐Allen, Gudiño, Korpelainen, & Long, 2011; Hedenäs et al., 2014; Heinrichs et al., 2015; Renner et al., 2013; Vanderpoorten, Patiño, Dirkse, Blockeel, & Hedenäs, 2015; Vigalondo et al., 2016). These studies identified numerous morphologically not or weakly differentiated bryophyte species of which many have rather narrow ranges.

Prior to the advent of DNA‐based investigations, many bryophyte species were considered to have broad, often intercontinental ranges equivalent to the ranges of angiosperm genera (Shaw, 2001; Vanderpoorten, Gradstein, Carine, & Devos, 2010). The intercontinental distributions of these species were interpreted as a vicariant pattern within species of ancient origin (Schuster, 1983) some of which were thought to date back to the Jurassic (Stotler & Crandall‐Stotler, 1974). However, inferences of Mesozoic ages of bryophyte species have been contradicted by DNA‐based divergence time estimates that have identified crown‐group diversification events within the Cenozoic in many lineages (Cooper, Henwood, & Brown, 2012; Feldberg et al., 2014; Laenen et al., 2014; Wilson, Heinrichs, Hentschel, Gradstein, & Schneider, 2007). Divergence time estimates suggest long‐distance dispersal (LDD) is more likely than vicariance as the process resulting in extant intercontinental ranges (Devos & Vanderpoorten, 2009; Dong et al., 2012; Hartmann, Wilson, Gradstein, Schneider, & Heinrichs, 2006; Scheben, Bechteler, Lee, Pócs, Schäfer‐Verwimp, & Heinrichs, 2016; Sun, He, & Glenny, 2014). Divergence time estimates also suggested an important role of angiosperm‐dominated forests in shaping the diversity of epiphyllic cryptogams (Feldberg et al., 2014).

**Figure 1:**
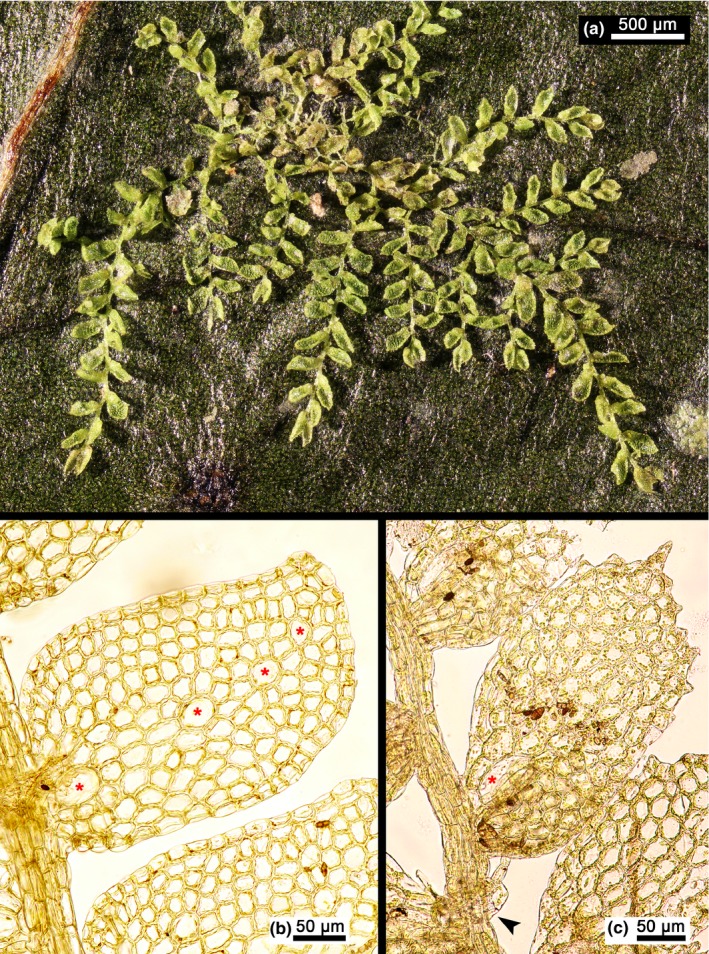
Images of two species of *Leptolejeunea*. (a) Habitus of dried herbarium specimen of *Leptolejeunea convexistipa* showing epiphyllous growth on a fern leaf. (b) Leaf of *Leptolejeunea epiphylla* with four ocelli in a broken row indicated by red stars. (c) Part of shoot of *Leptolejeunea convexistipa* focusing on a leaf with one basal ocellus (red star). Note the characteristic underleaf of the genus *Leptolejeunea* at the bottom left corner (black arrowhead)

*Leptolejeunea* (Spruce) Steph. is a pantropical genus of nearly exclusively epiphyllous leafy liverworts that grow in lowland and lower montane rainforests, occasionally also in high montane rainforests up to ca. 3,000 m (Bischler, 1969). The genus includes both local endemics (Shu, Zhu, & Pócs, 2016) and intercontinentally distributed species such as *L. elliptica*,* L. epiphylla*, and *L. maculata* (Grolle, 1976; Pócs & Lye, 1999; Schuster, 1980; Zhu & So, 2001). *Leptolejeunea* is characterized by its minute size, deeply bilobed underleaves with two widely divergent and subulate lobes, elliptic to narrowly obovate leaf lobes often with dentate margins, the presence of one to several ocelli in leaf lobes, and vegetative reproduction by cladia (Figure 1). Several species show a tendency for dry leaves to become elevated and produce monoterpenes that emit a strong fragrance (Gradstein, Churchill, & Salazar‐Allen, 2001) meaning the genus can be readily identified even in the field; yet identification of species is notoriously difficult. Söderström et al. (2016) accepted 48 species but indicated knowledge problems or serious doubts about the taxonomic value of many. An earlier study estimated global diversity at 25 species (Gradstein et al., 2001). So far, only a few accessions have been included in molecular phylogenetic studies (Ahonen, Muonen, & Piippo, 2003; Heinrichs et al., 2014; Wilson, Gradstein, Schneider, & Heinrichs, 2007). Results from these studies rejected a previously hypothesized close relationship between *Leptolejeunea* and *Drepanolejeunea* based on shared underleaf shape and the presence of ocelli in leaves of both genera (Gradstein, 2013), and resolved *Leptolejeunea* in a relatively isolated position within Lejeuneaceae subf. Lejeuneoideae (Heinrichs et al., 2014). Lejeuneaceae subtribe Leptolejeuneinae was established as a result to accommodate *Leptolejeunea* (Heinrichs et al., 2014). However, molecular phylogenetic investigations conducted to date have not improved current morphology‐based species concepts nor resolved biogeographic patterns.

Currently, in contradiction to more traditional views of morphological species, widespread *Leptolejeunea* species are believed to be the result of recent LDD out of Asia, a hypothesis promoted by Schuster (1983: 618): “taxa such as *Leptolejeunea elliptica*… have shown dispersal, clearly in geologically recent times, well out from Asia into the Pacific, to South America, Central and southern North America.” Here, we extend the sampling of Heinrichs et al. (2014) and test previous hypotheses on origins and extant distribution of *Leptolejeunea* species. We provide evidence for a Cenozoic origin of the *Leptolejeunea* crown group and reject pantropical species ranges.

## 2 Materials and Methods

### 2.1 Taxon sampling, DNA extraction, PCR amplification, sequencing, and alignment

Tissue for DNA extraction was isolated from *Leptolejeunea* specimens from the herbaria EGR, GOET, SP, and Schäfer‐Verwimp (SV). Specimens were revised based on literature and considering results from phylogenetic analyses. Total genomic DNA was isolated using the Invisorb Spin Plant Mini Kit (Stratec Molecular GmbH, Berlin, Germany). Four markers were amplified: the nuclear ribosomal internal transcribed spacer region (ITS1‐5.8S‐ITS2), the chloroplast *rbc*L gene, the *trn*L‐*trn*F region, and the *psb*A gene together with the *psb*A‐*trn*H intergenic spacer. PCR amplification of the first three markers follows Bechteler, Lee, Schäfer‐Verwimp, Pócs, et al. (2016). The *psb*A/*psb*A‐*trn*H region was amplified using the PCR program and primers (*trn*K2F, 510F, 576R, *trn*HR) described in Forrest and Crandall‐Stotler (2004). This protocol was modified as follows: 0.4 μL of MyTaq Polymerase (Bioline Reagents Ltd., UK), 11 μL of reaction buffer, 1 μL of upstream primer, 1 μL of downstream primer, and 1 μL of template DNA. The mix was filled up with double‐distilled water to a total volume of 50 μL. Representatives of *Pycnolejeunea* and *Xylolejeunea* were chosen as outgroups following phylogenetic hypotheses of Wilson, Gradstein, et al. (2007), Bechteler, Lee, Schäfer‐Verwimp, Pócs, et al. (2016) and Bechteler, Lee, Schäfer‐Verwimp, Renner, et al. (2016). Corresponding sequences were downloaded from GenBank (http://www.ncbi.nlm.nih.gov/genbank/), in addition to published sequences of *Leptolejeunea*. The resulting dataset comprised 66 specimens of *Leptolejeunea* and three specimens each of *Pycnolejeunea* and *Xylolejeunea* (Table 1). All sequences were aligned manually with bioedit 7.1.3.0 (Hall, 1999), and ambiguous sites were excluded.

**Table 1:** Taxa used in this study, including information about the geographical origin, voucher details, as well as GenBank accession numbers. Accession numbers in bold were obtained from GenBank

Taxon	Origin	Collector, voucher number, and herbarium	GenBank accession numbers			
			*rbc*L	*trn*LF	*psb*A	nrITS
*Leptolejeunea amphiophthalma* Zwickel	Malaysia	Pócs et al. 13168/AA (EGR)	KX808754	KX808806	KY006551	KX808704
*Leptolejeunea astroidea* (Mitt.) Steph.	Príncipe Island	Shevock 40015A (EGR)	KX808792	KX808851	KY006539	KX808742
*L. astroidea*	Uganda	Pócs et al. 97108/O (EGR)	KX808791	KX808850	–	KX808741
*Leptolejeunea balansae* Steph.	Malaysia	Pócs et al. 13184/F (EGR)	KX808777	KX808832	KY006538	KX808725
*Leptolejeunea brasiliensis* Bischl.	Brazil (I)	Peralta & Carmo 14222 (SP)	KX808758	KX808810	KY006502	KX808708
*L. brasiliensis*	Brazil (II)	Yano 28424 (SP)	KX808756	KX808808	KY006500	KX808706
*L. brasiliensis*	Brazil (III)	Peralta & Guiglota 13863 (SP)	KX808757	KX808809	KY006501	KX808707
*Leptolejeunea convexistipa* Bischl.	Dominican Republic	Schäfer‐Verwimp & Verwimp 27206/B (SV)	KX808800	–	KY006540	–
*L. convexistipa*	Ecuador (I)	Schäfer‐Verwimp 24419/C (SV)	KX808799	–	–	KX808748
*L. convexistipa* published as *elliptica* (Lehm. & Lindenb.) Schiffn.	Ecuador (II)	Wilson et al. 04‐18 (GOET)	**DQ983698**	–	**EF011862**	**DQ987375**
*L. convexistipa*	Ecuador (III)	Schäfer‐Verwimp et al. 24407/E (SV)	KX808798	KX808856	KY006533	KX808747
*L. convexistipa*	Panama (I)	Schäfer‐Verwimp & Verwimp 30861 (JE)	**KF954161**	**KF954151**	–	**KF954154**
*L. convexistipa*	Panama (II)	Schäfer‐Verwimp & Verwimp 30937/A (SV)	KX808801	KX808857	KY006534	KX808749
*Leptolejeunea dapitana* Steph.	Malaysia (I)	Pócs et al. 13160/Q (EGR)	KX808772	KX808824	KY006513	KX808719
*L. dapitana*	Malaysia (II)	Pócs et al. 13160/L (EGR)	KX808771	KX808823	KY006512	KX808718
*L. dapitana*	Vietnam	Luong TP211‐004b (EGR)	KX808770	KX808822	KY006511	KX808717
*Leptolejeunea elliptica* (Lehm. & Lindenb.) Schiffn.	Dominican Republic	Pócs & Pócs 03157/AB (GOET)	KX808795	KX808854	KY006532	KX808744
*L. elliptica*	Ecuador (I)	Schäfer‐Verwimp & Nebel 32794 (SV)	KX808794	KX808853	KY006531	KX808743
*L. elliptica*	Ecuador (II)	Schäfer‐Verwimp & Nebel 32834/A (SV)	KX808797	–	KY006552	KX808746
*L. elliptica*	Guadeloupe	Schäfer‐Verwimp & Verwimp 22518 (SV)	KX808793	KX808852	KY006541	–
*L. elliptica*	Jamaica	Schäfer‐Verwimp 34834/E (SV)	KX808796	KX808855	KY006549	KX808745
*Leptolejeunea epiphylla* (Mitt.) Steph.	Cambodia	Pócs s.n. (SV)	KX808765	KX808817	KY006546	KX808703
*L. epiphylla*	Malaysia (I)	Pócs et al. 13172/F (EGR)	KX808764	KX808816	KY006550	KX808713
*L. epiphylla*	Malaysia (II)	Schäfer‐Verwimp & Verwimp 19081 (JE)	**KF954163**	–	–	**KF954156**
*L. epiphylla*	Mayotte	Pócs et al. 9288/AA (EGR)	–	KX808818	KY006508	KX808714
*L. epiphylla*	Príncipe Island (I)	Shevock 40133 (SV)	KX808767	KX808819	KY006509	KX808715
*L. epiphylla*	Príncipe Island (II)	Shevock 42132 (EGR)	KX808768	KX808820	KY006545	KX808702
*L. epiphylla*	Indonesia, Sumatra	Schäfer‐Verwimp & Verwimp 24962/A (SV)	KX808769	KX808821	KY006510	KX808716
*L. epiphylla*	Thailand	Schäfer‐Verwimp 16245 (SV)	KX808766	–	–	KX808701
*Leptolejeunea exocellata* (Spruce) A.Evans	Argentina	Schäfer‐Verwimp & Verwimp 9330 (GOET)	KX808760	KX808812	KY006504	KX808700
*L. exocellata*	Dominican Republic (I)	Schäfer‐Verwimp & Verwimp 27018/A (SV)	KX808763	KX808815	KY006507	KX808712
*L. exocellata*	Dominican Republic (II)	Schäfer‐Verwimp & Verwimp 27197/A (SV)	KX808761	KX808813	KY006505	KX808710
*L. exocellata*	Dominican Republic (III)	Schäfer‐Verwimp & Verwimp 27215/C (SV)	KX808762	KX808814	KY006506	KX808711
*L. exocellata*	Ecuador	Schäfer‐Verwimp et al. 24407/C (SV)	KX808759	KX808811	KY006503	KX808709
*Leptolejeunea foliicola* Steph.	Indonesia, Bali	Schäfer‐Verwimp & Verwimp 16689/E (SV)	–	KX808843	–	KX808734
*L. foliicola*	Malaysia (I)	Schäfer‐Verwimp & Verwimp 18903/C (SV)	KX808785	KX808842	KY006525	KX808733
*L. foliicola*	Malaysia (II)	Schäfer‐Verwimp & Verwimp 18976 (SV)	KX808786	KX808844	KY006526	KX808735
*Leptolejeunea maculata* (Mitt.) Schiffn.	Malaysia (I)	Pócs et al. 13171/G (EGR)	KX808782	KX808839	KY006523	KX808731
*L. maculata*	Malaysia (II)	Pócs et al. 13168/AE (EGR)	KX808783	KX808840	KY006542	–
*L. maculata*	Malaysia (III)	Pócs et al. 13167/AM (EGR)	–	KX808837	KY006521	KX808729
*L. maculata*	Malaysia (IV)	Schäfer‐Verwimp & Verwimp 18599/A (SV)	KX808781	KX808838	KY006522	KX808730
*Leptolejeunea moniliata* Steph.	Guadeloupe	Schäfer‐Verwimp & Verwimp 22117/A (SV)	KX808755	KX808807	KY006499	KX808705
*Leptolejeunea radicosa* (Nees ex Mont.) Grolle	Dominica	Schäfer‐Verwimp & Verwimp 17723/C (JE)	**KF954165**	–	–	**KF954158**
*L. radicosa*	Guadeloupe (I)	Schäfer‐Verwimp & Verwimp 22305/A (SV)	KX808804	KX808860	KY006537	KX808753
*L. radicosa*	Guadeloupe (II)	Schäfer‐Verwimp & Verwimp 22417/E (SV)	KX808803	KX808859	KY006536	KX808751
*L. radicosa*	Guadeloupe (III)	Schäfer‐Verwimp & Verwimp 22414/D (SV)	KX808805	–	–	KX808752
*L. radicosa*	Panama	Schäfer‐Verwimp & Verwimp 30795 (SV)	KX808802	KX808858	KY006535	KX808750
*Leptolejeunea schiffneri* Steph.	Malaysia	Schäfer‐Verwimp & Verwimp 18619/A (SV)	KX808773	KX808826	KY006548	–
*L. schiffneri*	Mayotte (I)	Pócs et al. 05106/BK (SV)	KX808776	KX808829	KY006516	KX808722
*L. schiffneri*	Mayotte (II)	Pócs et al. 05105/E (EGR)	–	KX808830	KY006544	–
*L. schiffneri*	Indonesia, Sumatra (I)	Schäfer‐Verwimp & Verwimp 25233/B (SV)	KX808775	KX808828	KY006515	KX808723
*L. schiffneri*	Indonesia, Sumatra (II)	Schäfer‐Verwimp & Verwimp 25233/B1 (SV)	KX808774	KX808827	KY006547	KX808721
*L. schiffneri*	Indonesia, Sumatra (III)	Schäfer‐Verwimp & Verwimp 25228 (SV)	–	KX808825	KY006514	KX808720
*Leptolejeunea* spec.	Thailand (I)	Chantanaorrapint 1352 (EGR)	–	KX808831	KY006517	KX808724
*Leptolejeunea* spec.	Thailand (II)	Schäfer‐Verwimp & Verwimp 16177 (SV)	KX808779	KX808835	KY006520	KX808728
*Leptolejeunea subacuta* Steph. ex A.Evans published as *elliptica* (Lehm. & Lindenb.) Schiffn.	China	Koponen et al. 50179 (H)	**AY125939**	**AY144480**	–	–
*L. subacuta*	Laos	Peregovits NoLaos/8 (EGR)	KX808789	KX808847	KY006498	KX808737
*L. subacuta*	Japan, Ryukyu Islands	Yamaguchi 15722 (GOET)	KX808787	KX808845	KY006527	KX808736
*L. subacuta*	Thailand (I)	Schäfer‐Verwimp & Verwimp 23785/C (SV)	–	KX808848	KY006529	KX808739
*L. subacuta*	Thailand (II)	Schäfer‐Verwimp & Verwimp 23791/B (SV)	KX808790	KX808849	KY006530	KX808740
*L. subacuta*	Thailand (III)	Schäfer‐Verwimp & Verwimp 23834/A (SV)	KX808788	KX808846	KY006528	KX808738
*Leptolejeunea* cf. *subrotundifolia* Herzog	Madagascar	Pócs & Szabo 9875/AZ (EGR)	KX808780	KX808836	KY006543	–
*Leptolejeunea* cf. *subrotundifolia*	Thailand	Pócs & Somadee 1228/C (EGR)	KX808784	KX808841	KY006524	KX808732
*Leptolejeunea vitrea* (Nees) Schiffn.	Malaysia (I)	Dürhammer D148 (JE)	**KF954164**	**KF954152**	–	**KF954157**
*L. vitrea*	Malaysia (II)	Pócs et al. 13175/O (EGR)	–	KX808833	KY006518	KX808726
*L. vitrea*	Philippines	Schumm & Schwarz 6425 (SV)	KX808778	KX808834	KY006519	KX808727
*Pycnolejeunea densistipula* (Lehm. & Lindenb.) Steph.	Ecuador	Schäfer‐Verwimp & Preussing 23368 (GOET)	**AY548075**	**DQ987400**	**EF011774**	**DQ987294**
*Pycnolejeunea macroloba* (Nees & Mont.) Schiffn.	Brazil	Yano 32740 (M)	**KJ408354**	**KJ408378**	–	**KJ408329**
*Pycnolejeunea sphaeroides* (Sande Lac.) J.B.Jack & Steph.	Malaysia	Schäfer‐Verwimp & Verwimp 18615/B (M)	**KJ408355**	**KJ408379**	–	**KJ408330**
*Xylolejeunea crenata* (Nees & Mont.) X.L.He & Grolle	Brazil	Schäfer‐Verwimp 11225 (GOET)	**DQ983740**	**DQ987443**	**EF011822**	**DQ987341**
*X. crenata*	Ecuador	Schäfer‐Verwimp & Nebel 32827/A (M)	**KJ408356**	**KJ408382**	–	**KJ408333**
*Xylolejeunea grolleana* (Pócs) X.L.He & Grolle	Madagascar	Pócs & Szabó 9878/EM (EGR)	**KT626911**	**KT626928**	–	**KT626892**

### 2.2 Phylogenetic analyses

Maximum‐likelihood (ML) analyses were conducted using RAxML 8.2.4 (Stamatakis, 2014). The best fit models of evolution selected by jmodeltest 2 (Darriba, Taboada, Doallo, & Posada, 2012) under the Akaike information criterion (AIC; Akaike, 1973) were as follows: TIM3+I+G for *rbc*L, TPM1uf+G for *trn*L‐*trn*F, TIM3+I+G for *psb*A/*psb*A‐*trn*H, and TIM3+I+G for nrITS1‐5.8S‐ITS2. These were not available in RaxML so the best fitting overparameterized model, GTR+G, was used for all markers (Posada, 2008). First, all markers were analyzed separately on the CIPRES Science Gateway (Miller, Pfeiffer, & Schwartz, 2010) using the “thorough ML” option, and an additional analysis was carried out for a combined chloroplast DNA dataset. Clades with bootstrap values (BP) of 70%–94% were regarded as moderately supported and those with BP ≥95% as strongly supported (Erixon, Svennblad, Britton, & Oxelman, 2003). No strongly supported topological contradictions between single markers or the nuclear and plastid datasets were detected. Accordingly, all matrices were concatenated, resulting in an alignment of 3,694 nucleotide positions. Ten thorough ML searches in combination with multiparametric bootstrapping using the autoMRE function (Pattengale, Alipour, Bininda‐Emonds, Moret, & Stamatakis, 2010) were conducted.

Bayesian inference was undertaken with mrbayes 3.2.6 (Ronquist & Huelsenbeck, 2003) using a partition for each marker and a GTR substitution model with rate of invariable sites and gamma rate heterogeneity as recommended by jmodeltest 2. Two metropolis‐coupled Markov chain Monte Carlo (MCMC) analyses, including three heated chains and one cold chain, were run for 10 million generations, sampled every 1,000 generations. TRACER 1.6 (http://tree.bio.ed.ac.uk/software/tracer/) was used to check for convergence and stationarity, and an average standard deviation (*SD*) of split frequency below 0.01 indicated a sufficiently long run. The initial 25% of sampled trees were discarded as burn‐in. The remainder were summarized with treeannotator 1.8.2 (Drummond, Suchard, Xie, & Rambaut, 2012), and the resulting maximum clade credibility (MCC) tree was visualized using figtree 1.4.2 (http://tree.bio.ed.ac.uk/software/figtree/). BPP values ≥0.95 were regarded as good support (Larget & Simon, 1999).

### 2.3 Divergence time estimates and biogeography

Dating analyses were performed using BEAST 1.8.2 (Drummond et al., 2012) using the same partitioning scheme and substitution models as the mrbayes analyses. An ultrametric starting tree without time scale was generated by setting the ingroup monophyletic, using linked trees over all partitions, 60 million generations and sampling every 6,000 generations. An uncorrelated log‐normal (UCLN) relaxed clock and a birth–death prior accounting for incomplete sampling (Stadler, 2009) were used. The result was inspected in TRACER, and ESS values >200 indicated good mixing of the MCMC and a sufficient number of generations. A MCC tree was generated with treeannotator 1.8.2 after discarding the first 10% of trees as burn‐in and visualized in figtree. This tree was used as a starting tree for subsequent divergence time estimates. Again, the ingroup was constrained as monophyletic, trees were linked over all partitions, and this analysis ran for 100 million generations sampling every 10,000 generations. As no *Leptolejeunea* fossils are known, a plastid genome substitution rate of 5 × 10^−4^ subst./sites/my (Palmer, 1991; Villarreal & Renner, 2012) was used for the three chloroplast markers with a *SD* of 1 × 10^−4^ and a normal prior distribution. For the nrITS region, a substitution rate of 1.35 × 10^−3^ subst./sites/my was adopted from Les, Crawford, Kimball, Moody, & Landolt (2003). A normal prior distribution in combination with the truncate option and upper and lower bounds of 0.4–8.3 × 10^−3^ subst./sites/my was implemented to allow the rate to vary over the large spectrum of reported nrITS rates (Kay, Whittall, & Hodges, 2006; Villarreal & Renner, 2014). The stepping‐stone sampling in BEAST (Baele, Li, Drummond, Suchard, & Lemey, 2013; Baele et al., 2012; Xie, Lewis, Fan, Kuo, & Chen, 2011) and the Bayes factor (Kass & Raftery, 1995) were used to compare between pure‐birth (Yule), birth–death, and birth–death incomplete sampling tree priors, as well as an UCLN relaxed clock and a strict clock. This resulted in choosing a birth–death incomplete sampling prior in combination with a UCLN relaxed clock model. Log marginal‐likelihood values and Bayes factor values are shown in Table 2. Results of the BEAST run were examined in TRACER, summarized in treeannotator by median branch lengths, and visualized in figtree.

**Table 2:** Marginal‐likelihood estimations using stepping‐stone sampling in BEAST and ln Bayes factor calculation resulting in an uncorrelated log‐normal (UCLN) relaxed clock model and a birth–death tree prior accounting for incomplete sampling (BDincompl.) for the *Leptolejeunea* dataset

	Model 1	BDincompl., UCLN	BD, UCLN	Yule, UCLN	BDincompl., strict clock
**Model 2**	Log marginal likelihood	−17,905.64	−17,911.82	−17,940.59	−17,945.11
BDincompl., UCLN	−17,905.64	0.00	−6.17	−34.95	−39.46
BD, UCLN	−17,911.82	6.17	0.00	−28.78	−33.29
Yule, UCLN	−17,940.59	34.95	28.78	0.00	−4.51
BDincompl., strict clock	−17,945.11	39.46	33.29	4.51	0.00

Afromadagascar, Asia–Australasia, and tropical–subtropical America were chosen as putative areas of endemism, and each specimen was assigned to one of these regions according to the label information. Ancestral areas of distribution were reconstructed using maximum parsimony criteria as implemented in mesquite 3.1 (Maddison & Maddison, 2016) based on the MCC topology from the divergence time analysis. In addition, the R‐package biogeoBEARS (Matzke, 2013a, 2013b, 2014) was employed to infer the ancestral history of *Leptolejeunea*. This likelihood‐based method implements the LAGRANGE DEC model (Ree & Smith, 2008), DIVA (dispersal‐vicariance analysis; Ronquist, 1997), and BayArea (Landis, Matzke, Moore, & Huelsenbeck, 2013), each of which can be extended with an additional free parameter *j* accounting for founder‐event speciation. To obtain the recommended operational taxonomic units consisting of monophyletic populations and not individual specimens, specimens of one species with the same putative area of endemism were merged together into a single terminal using the R‐script provided on the biogeoBEARS webpage (http://phylo.wikidot.com/example-biogeobears-scripts#pruning_a_tree). All six models were compared using likelihood values, the AIC, and the AIC corrected for small sample size (AIC_c_) (Matzke, 2014). The maximum number of areas was set to three to account for the assumed pantropical ranges of *Leptolejeunea* species (Grolle, 1976; Pócs, 2012; Pócs & Lye, 1999; Schuster, 1983).

### 2.4 Morphological investigation

Specimens were studied under a Carl Zeiss AxioScope A1 compound microscope equipped with a Canon 60D digital camera using transmitted or incident light. The *Leptolejeunea convexistipa* voucher Schäfer‐Verwimp 35198/A (M) and the *L. epiphylla* voucher Schäfer‐Verwimp 16245 (M) were digitized (Figure 1). All presented images are digitally stacked photomicrographic composites of up to 20 individual focal planes obtained using the software package HeliconFocus 6.7.1.

## 3 Results

### 3.1 Phylogeny

**Figure 2:**
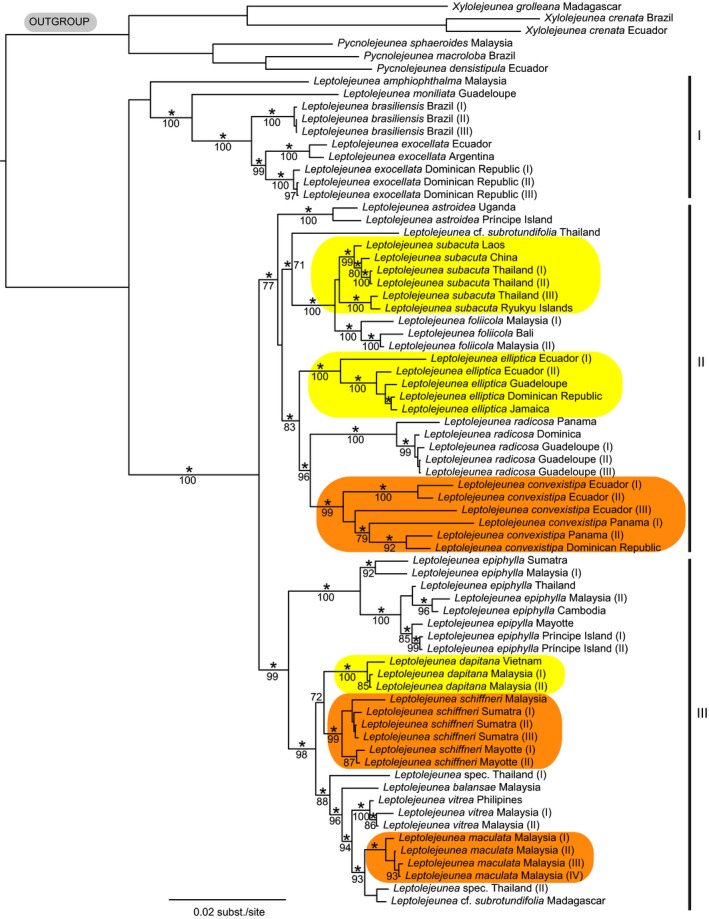
Majority rule consensus tree of trees recovered in stationary phase of Bayesian search. A star indicates a Bayesian Posterior probability >.97. Maximum‐likelihood bootstrap percentage values >70 are also shown at branches. Orange highlighted accessions were earlier considered to belong to *Leptolejeunea maculata*, and yellow highlighted accessions were earlier considered to belong to *L. elliptica*

*Leptolejeunea* splits into three main clades (labeled I, II, III) with clade I placed sister to the remainder of the genus (Figure 2). Clade I includes a Malaysian accession of *L. amphiophthalma* in an unsupported sister relationship to a robust Neotropical clade consisting of *L. moniliata*,* L. brasiliensis,* and *L. exocellata*. Three accessions of *L. brasiliensis* were placed sister to a clade with five accessions of *L. exocellata*. A clade with three accessions of *L. exocellata* from the Dominican Republic was placed sister to a clade with *L. exocellata* accessions from Argentina and Ecuador. Clade II achieved a BPP of 1.00 and a BP of 77 and included a lineage with accessions of *L. astroidea* from Uganda and Príncipe Island, a lineage with Asian accessions of *L. subacuta*,* L. *cf. *subrotundifolia* and *L. foliicola*, and a Neotropical lineage with accessions of *L. convexistipa*,* L. elliptica,* and *L. radicosa*. An accession of *L. radicosa* from Panama was placed sister to a clade with accessions from Dominica and Guadeloupe. Clade III comprised Paleotropical accessions (BPP 1.00, BP 99). *Leptolejeunea epiphylla* split into a clade with two accessions from Sumatra and Malaysia, and a clade with accessions from Cambodia, Malaysia, and Thailand in a sister relationship with accessions from Mayotte and Príncipe Island. The *L. epiphylla* clade was sister to a clade with accessions assigned to *L. dapitana*,* L. maculata*,* L. schiffneri*,* L. vitrea*,* L. balansae*,* L. *cf. *subrotundifolia,* and *L. *spec. indet. The *L. schiffneri* clade included an Asian lineage and a lineage with accessions from Mayotte. Representatives of other clade III species originated exclusively from Asia. Most species represented by multiple accessions achieved BPPs of 1.00 and BPs >98; *L. maculata* achieved a BPP of 0.99 and a BP of 62; the monophyly of *L. subacuta* was unsupported.

### 3.2 Divergence time estimates and biogeography

**Figure 3:**
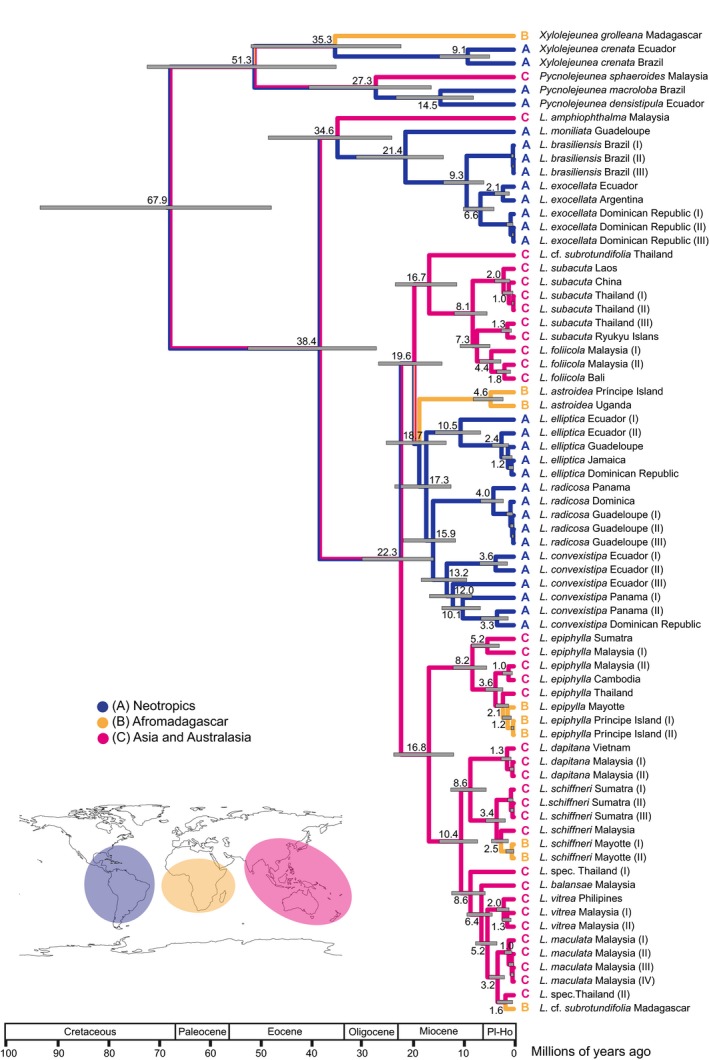
BEAST chronogram with 95% highest posterior density (HPD) intervals and branches colored according to the most parsimonious reconstruction of distributions of *Leptolejeunea*. Putative areas of endemism are indicated for every accession rather than morphospecies. Node ages ≥1 Ma are reported

**Figure 4:**
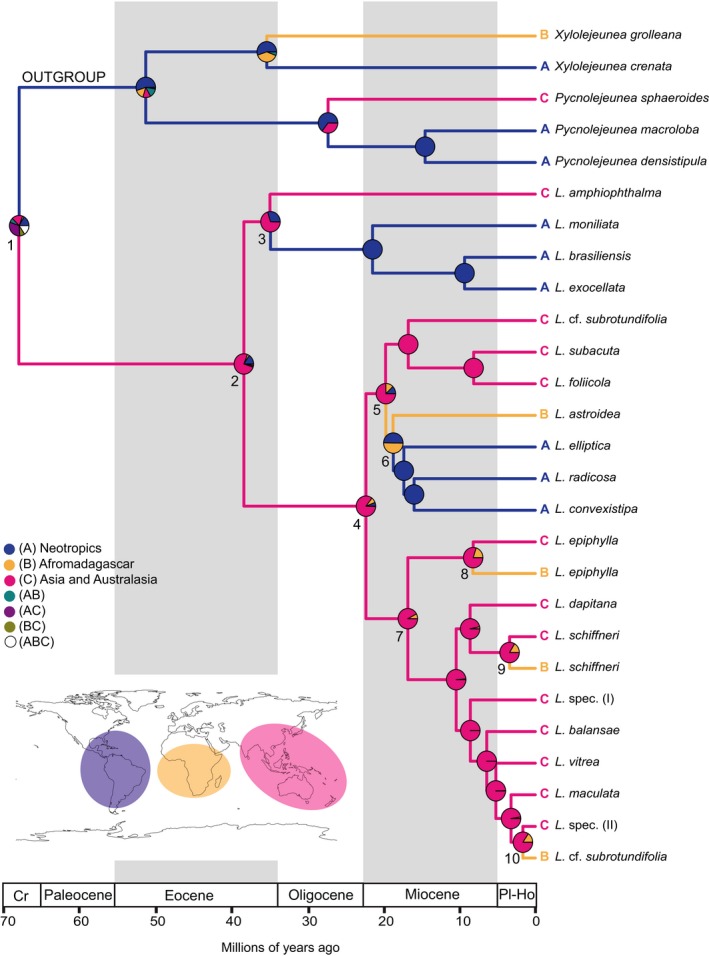
Result of the biogeo BEARS analysis of *Leptolejeunea* in combination with the modified BEAST chronogram. Circles at nodes represent probabilities for ancestral areas resulting from DIVALIKE analysis accounting for founder‐event speciation. See Table 4 for percent values. Branches are colored according to the most probable area for splits as indicated by biogeo BEARS

The divergence time analyses (Figure 3) provided evidence for a split between the outgroup and *Leptolejeunea* in a time interval from the Early Eocene to the Late Cretaceous (67.9 Ma, 95% HPD: 47.9–93.7) and an Oligocene to Eocene (38.4 Ma, 95% HPD: 27.2–52.6) age of the *Leptolejeunea* crown group. Most of the species clades were established in the Miocene. The biogeoBEARS analyses favored a DIVALIKE+J model for the estimation of ancestral areas (Table 3), and results obtained with this model are shown in Figure 4 in combination with the modified BEAST chronogram. Estimated ancestral area probabilities for selected nodes are given in Table 4. The origin of *Leptolejeunea* is ambiguous, with the highest probability of an origin in Asia or the Neotropics. Similar results were achieved using maximum parsimony criteria (Figure 3). Neotropical–Paleotropical disjunctions occurred during the Miocene to Eocene. *Leptolejeunea epiphylla* and *L. schiffneri* originated in Asia and colonized African islands during the Plio‐Pleistocene.

**Table 3:** Results of the biogeoBEARS analyses favoring a DIVALIKE+J model, as shown in bold, according to model selection by log‐likelihood values (lnL), Akaike information criterion (AIC), and AIC corrected for small sample size (AIC_c_)

	lnL	*n*	*d*	*e*	*j*	AIC	AIC_c_
DEC	−42.11	2	0.008	0.003	0	88.22	88.72
DEC+J	−24.95	3	10^−12^	10^−12^	0.15	55.89	56.94
DIVALIKE	−38.08	2	0.009	10^−12^	0	80.16	80.66
**DIVALIKE+J**	−**24.79**	**3**	**10** ^**−12**^	**10** ^**−12**^	**0.14**	**55.59**	**56.63**
BAYAREALIKE	−54.58	2	0.009	0.03	0	113.2	113.7
BAYAREALIKE+J	−25.77	3	10^−7^	10^−7^	0.14	57.54	58.58

*n*, number of parameters; *d*, rate of dispersal; *e*, rate of extinction; *j*, relative probability of founder‐event speciation.

**Table 4:** Estimated ancestral area probabilities for selected nodes obtained from the biogeoBEARS analysis of *Leptolejeunea* rounded in percent. Node numbers are displayed in Figure 4. Areas are coded as follows: A, Neotropics; B, Afromadagascar; C, Australasia; AB, AC, BC, ABC are combinations of these areas

Node	Estimated ancestral area (DIVALIKE+J)
1	A 17, B 3, C 18, AB 7, AC 31, BC 8, ABC 16
2	A 16, B 3, C 76, AC 3, BC 3
3	A 30, C 70
4	A 6, B 8, C 85, BC 1
5	A 12, B 13, C 75
6	A 50, B 50
7	B 8, C 92
8	B 20, C 80
9	B 16, C 84
10	B 16, C 84

## 4 Discussion

### 4.1 Bryophyte species in the molecular age

Although intercontinentally disjunct bryophyte species often form monophyla (Heinrichs et al., 2010; Vigalondo et al., 2016), accessions from different continents are often resolved in sister clades (Heinrichs et al., 2011). This pattern of geographically structured phylogenetic relationships suggests gene flow and interbreeding between populations on different continents has ceased, and this may be confirmed by detailed study (Medina, Lara, Goffinet, Garilleti, & Mazimpaka, 2013). Other studies point to the polyphyly of supposedly intercontinentally distributed species (Huttunen & Ignatov, 2010; Renner, 2014) and indicate that monophyletic bryophyte species often have restricted ranges (Medina, Lara, Goffinet, Garilleti, & Mazimpaka, 2012; Medina et al., 2013; Renner et al., 2013). That patterns of phylogenetic and morphological diversification are often decoupled in bryophytes is now well recognized, and many instances of morphologically cryptic species complexes have been documented (Baczkiewicz & Buczkowska, 2016; Kyrkjeeide, Hassel, Flatberg, Shaw, Yousefi, et al., 2016; Odrzykoski & Szweykowski, 1991; Ramaiya et al., 2010; Shaw, Boles, & Shaw, 2008). However, the prevalence of morphologically cryptic divergence, and the number of species resulting from such events, remains unknown. Species circumscription based on morphology may overlook two important features: firstly, the existence of higher phylogenetic diversity than suggested by patterns of morphological variation and secondly, higher geographic structuring than suggested by the distribution of morphological variation (Medina et al., 2013; Ramaiya et al., 2010; Renner, Brown, & Wardle, 2011; Renner et al., 2013).

### 4.2 *Leptolejeunea* species ranges and taxonomy

Our study contradicts hypothesized pantropical ranges for two *Leptolejeunea* species (Figure 2, note highlighted specimens) and supports the hypothesis of Shaw (2001) that morphological uniformity of bryophytes often belies a complex genetic structure. According to our sampling, *L. elliptica* is restricted to the Neotropics rather than representing a pantropical species (Pócs, 2012; Schuster, 1980). Paleotropical accessions that were earlier assigned to *L. elliptica* are placed in separate lineages and have been revised to *L. dapitana* and *L. subacuta* (Figure 2). The supposedly pantropical *L. maculata* (Grolle, 1976; Pócs & Lye, 1999) forms three independent lineages (Figure 2). Asian *L. maculata* s.str. is placed in main clade III, together with a Paleotropical lineage here identified as *L. schiffneri*. Neotropical accessions of *L. maculata* belong to main clade II and have been identified as *L. convexistipa*. Such findings have frequently been explained as instances of cryptic or near cryptic speciation (Shaw, 2001); however, molecular topologies may allow revision of morphological evidence and the identification of morphological character states supporting the different lineages (Forrest et al., 2011; Heinrichs et al., 2015; Renner et al., 2013). Revision of *Leptolejeunea* specimens is challenging as the taxonomy of this genus relies heavily on the number and distribution of ocelli in the leaves, that is, specialized cells containing only a single large rather than several small oil bodies (He & Piippo, 1999). These often disappear from herbarium specimens. Exceptionally large or small leaf cells in herbarium specimens may be indicative of ocelli; however, ocelli sharing the size of the surrounding leaf cells may not be recognizable in dried materials. A thorough revision of *Leptolejeunea* thus needs to be based on the investigation of living plants from all parts of the range and sequencing of a comprehensive number of specimens including types or topotypes. New sources of species circumscribing characters also need to be sought. Such work is beyond the scope of this study; however, our data facilitate discrimination between alternative interpretations of species circumscription and to reconstruct the distribution of the main clades. Our data also support the finding of Renner (2015) that morphologically similar leafy liverworts may be placed in different main lineages, despite considerable morphological overlap. Accessions originally assigned to the same species were resolved in different main clades, and the supposedly closely related species *L. brasiliensis* and *L. elliptica* (Schuster, 1980) were resolved in main clade I or II (Figure 2). Phylogenies of Lejeuneaceae genera often show a geographical pattern related to the distribution of lineages rather than a morphological pattern. Examples include the genera *Lejeunea* (Heinrichs et al., 2013) and *Diplasiolejeunea* (Dong et al., 2012) which exhibit separation into predominantly Neotropical and predominantly Paleotropical lineages. A similar situation manifests in *Leptolejeunea*.

### 4.3 Divergence time estimates, biogeography, and infraspecific variation

Our divergence time estimates suggest Cenozoic diversification of *Leptolejeunea* and contradict Gondwanan vicariance (Raven & Axelrod, 1974) as an explanation for the observed disjunctions. Establishment of the *Leptolejeunea* crown group in the Eocene accordances well with the appearance of humid megathermal angiosperm forests (Morley, 2011) which provided the preferred epiphyllous habitat of extant *Leptolejeunea* representatives. Cretaceous gymnosperm forests differed in structure and evaporated less water than tropical angiosperm forests (Boyce & Lee, 2010). Thus, they may not have hosted as diverse epiphyll communities or supported Lejeuneaceae representatives adapted to other niches than modern species (Feldberg et al., 2014). Similar evolutionary processes have been reconstructed for the genera *Lejeunea*,* Harpalejeunea*, and *Microlejeunea* based on molecular and fossil evidence (Heinrichs et al., 2016).

Our reconstruction failed to unambiguously identify the area of origin of *Leptolejeunea*; however, we need to consider the wide distribution of humid angiosperm forests in the Eocene including the northern “boreotropical” region (Morley, 2011). Lack of fossils and extant species precludes inference of a northern range for *Leptolejeunea*; however, the Eocene range of *Leptolejeunea* likely differed from the current distribution. Cooling during the Neogene (Zachos, Pagani, Sloan, Thomas, & Billups, 2001) may have resulted in range contraction and extinction in the north, and possibly the extinction of some early lineages. Caution interpreting biogeographical reconstructions utilizing standard substitution rates is always required; however, our chronogram suggests either lower speciation or higher extinction rates during the early Oligocene cooling phase (Liu et al., 2009), and the establishment of extant *Leptolejeunea* species predominantly in the Miocene. This pattern could relate to a Miocene reorganization of tropical forests. Miocene origins for extant diversity have also been observed in mosses (Lewis, Rozzi, & Goffinet, 2014; Shaw et al., 2010) and leptosporangiate ferns (Schneider et al., 2010; Wei et al., 2015). The age of the oldest Neotropical–Paleotropical disjunctions could relate to boreotropical migration (Davis, Bell, Matthews, & Donoghue, 2002; Le Péchon et al., 2016) although a thorough reconstruction is precluded by the lack of fossils. Miocene disjunctions are better explained by LDD, as are the island occurrences of several species. Liverworts have dispersed to the African continent and associated islands from both the Neotropics and Asia (Feldberg et al., 2007; Heinrichs et al., 2005). Both biogeographical analyses (Figures 3 and 4) provide evidence for an Asian origin of the African accessions of *L. epiphylla*,* L. schiffneri,* and *L. *cf. *subrotundifolia,* whereas the origin of the African *L. astroidea* remains unclear. African taxa nesting in Asian clades have also been described for ferns (Hennequin, Hovenkamp, Christenhusz, & Schneider, 2010; Janssen, Kreier, & Schneider, 2007) and angiosperms (Kulju, Sierra, Draisma, Samuel, & van Welzen, 2007; Li, Dressler, Zhang, & Renner, 2009; Richardson, Chatrou, Mols, Erkens, & Pirie, 2004). Monsoon trade winds were proposed as dispersal agent from Asia to Africa (Li et al., 2009) and could also be responsible for the observed pattern in *Leptolejeunea*. Alternatively, animal‐mediated dispersal may contribute to current disjunctions. At small spatial scales, millipedes have been demonstrated to move gemmae of species of the moss genus *Calymperes* (Zona, 2013). Larger animals that move over correspondingly larger spatial scales may also transport propagules and plant fragments (Lewis et al., 2014). In New Zealand, the isolated occurrences of the tropical *Calymperes tenerum* are congruent with known visitation sites of the predominantly tropical black‐winged petrel (P. J. de Lange, personal communication). Seabirds are known to visit potential or actual breeding sites, even though visiting individuals may not nest there. To visit these sites, which are often forested, birds literally crash through the canopy to the ground, thus coming into close, vigorous contact with leaf and twig surfaces, providing ample opportunity for plant fragments to become deeply embedded within the bird's feather matrix. Seabirds roam widely during their nonbreeding season and routinely traverse oceans and have been known to traverse land masses bridging oceanic waterways.

The island occurrences provide evidence for the ability of *Leptolejeunea* species to disperse over long distances either by vegetative propagules (Laenen et al., 2016) or by spores (Van Zanten & Gradstein, 1988). However, successful LDD seems rare in *Leptolejeunea*, as indicated by the plurispecies clades being restricted to either the Neotropics or the Paleotropics, but also by the genetic variation within single morphospecies. Although our data support a narrower species concept and reinstatement of several putative synonyms, some species clades still have a considerable molecular variation, with initial splits in the late Miocene (Figure 3). Examples include a split between mainland South American *L. exocellata* and accessions from the West Indian Islands, splits within Asian *L. epiphylla*, and splits within Neotropical *L. convexistipa*. Considerable molecular variation related to a geographical rather than a morphological pattern has been observed for a larger number of liverworts (Fuselier et al., 2009; Heinrichs et al., 2015; Ramaiya et al., 2010) although it is still somewhat unclear whether this variation is in general indicative of genetically independent entities. Follow‐up studies should thus involve denser sampling and additional markers including microsatellites. Intercontinental gene flow has already been demonstrated for bryophytes, especially for holarctic species of the moss genus *Sphagnum* (Kyrkjeeide, Hassel, Flatberg, Shaw, Brochmann, et al., 2016; Shaw et al., 2014); however, the epiphyllous habitat of *Leptolejeunea* species in the understory of tropical forests may lower the LDD success rate compared to *Sphagnum* species which occur in open wetland systems.

### 4.4 Perspectives

Every disjunction has its first day; hence, we cannot generally reject intercontinental or even pantropical species ranges (Lewis et al., 2014). On the other hand, a growing body of evidence indicates that LDD occurs only infrequently in bryophytes and that it is thus often associated with speciation. The accumulation of genetic disparity in bryophytes is often not associated with the accumulation of a similar amount of morphological disparity (Baczkiewicz & Buczkowska, 2016; Ramaiya et al., 2010), although there are exceptions (Heinrichs, Gradstein, Groth, & Lindner, 2003). Lack of molecular support for morphology‐based supraspecific taxa such as sections and subgenera (Devos, Renner, Gradstein, Shaw, & Vanderpoorten, 2011) further complicates the understanding of bryophyte evolution and appropriate choice of ingroup representatives. A reliable reconstruction of the evolutionary history and biogeography of bryophytes thus needs to be based on comprehensive molecular phylogenies with complete population‐level sampling. Only such phylogenies will facilitate species identification and refined estimation of bryophyte global diversity and origins.

## Conflict of Interest

None declared.
